# Technetium-99m-labeled rituximab for use as a specific tracer of sentinel lymph node biopsy: a translational research study

**DOI:** 10.18632/oncotarget.9614

**Published:** 2016-05-26

**Authors:** Xuejuan Wang, Zhi Yang, Baohe Lin, Yan Zhang, Shizhen Zhai, Qichao Zhao, Qing Xie, Fei Liu, Xuedi Han, Jinfeng Li, Tao Ouyang

**Affiliations:** ^1^ Key Laboratory of Carcinogenesis and Translational Research (Ministry of Education/Beijing), Department of Nuclear Medicine, Peking University Cancer Hospital & Institute, Beijing Cancer Hospital, Beijing 100142, P. R. China; ^2^ Key Laboratory of Carcinogenesis and Translational Research (Ministry of Education/Beijing), Breast Cancer Center, Peking University Cancer Hospital & Institute, Beijing 100142, P. R. China

**Keywords:** sentinel lymph node, biopsy, CD20, rituximab, specific

## Abstract

**Purpose:**

We aimed to develop and translate a CD20-antigen-targeted radiopharmaceutical, Technetium-99 m-labeled (^99m^Tc) rituximab, for sentinel lymph node (SLN) detection.

**Methods:**

^99m^Tc-rituximab was synthesized and tested for stability in human serum. The binding affinity to CD20 was evaluated in Raji cells by flow cytometric analysis. Biodistribution and sentinel node mapping were carried out in bal b/c mice. Eighty-five patients with breast cancer participated in this study. Dynamic sentinel lymphoscintigraphy was first assessed in 12 patients before planar lymphoscintigraphy was assessed in a larger cohort. All patients underwent sentinel lymph node biopsy (SLNB), followed by axillary lymph node dissection.

**Results:**

The cell-binding study showed that ^99m^Tc-rituximab possessed compatible affinity to human CD20. In the mechanism study, ^99m^Tc-labeled anti-mouse CD20 monoclonal antibodies could bind to mouse CD20 and accumulate in the SLN with 2.62±1.25 % of the percentage of injected activity, which could be blocked by excessive unlabeled antibody. Low uptake of non-sentinel nodes and fast clearance from the injection site were observed in the mice. Sentinel nodes were identified in 82 of 85 breast cancer patients (96.5%) by lymphoscintigraphy and SLNB. The sensitivity, specificity, and accuracy were 96.8% (30/31), 100% (51/51), and 98.8% (81/82), respectively.

**Conclusion:**

^99m^Tc-rituximab, specifically binding to CD20, met most of the requirements of an ideal sentinel mapping agent for use in clinical settings.

## INTRODUCTION

The presence or absence of axillary node metastasis remains the most powerful prognostic feature when stratifying breast cancer patients according to the risk of relapse. Traditionally, lymph node status was established by axillary lymph node dissection, but this approach changed with the development of sentinel lymph node biopsy (SLNB) [1, 2]. The sentinel lymph node (SLN) is defined as the first regional lymph node to receive lymphatic drainage from a primary malignant tumor and is the first node to which metastatic cells are likely to anchor [3]. The introduction of SLNB revolutionized the surgical management of breast cancer, providing a minimally invasive means to stage clinically negative regional nodes and identify patients with microscopic nodal involvement for early therapeutic intervention [4].

The existing standard SLNB method is a dual technique involving injection of a radiotracer and blue dye into either the interstitial breast tissue around the tumor or the periareolar tissue [5]. The radiotracer, normally labeled by technetium 99m (^99m^Tc), is first used preoperatively to determine the location of SLN and is then used intraoperatively to guide the dissection to the SLN, stained by the blue dye. Although as many as 60% of an estimated 500,000 patients have access to SLNB in developed countries, this figure falls to 5% in China and is even lower in the rest of the world [6]. One barrier to standardization is the fact that neither the blue dye nor any of the ^99 m^Tc-labeled agents have been approved by China Food and Drug Administration (CFDA) for SLNB.

The ideal radiopharmaceutical for lymphatic mapping should have several properties: it should be standardized and require minimal preparation or modification before injection; it should be promptly absorbed by the lymphatics and quickly transported to the first-echelon nodes; it should have sustained accumulation in the SLNs, in high amounts, and provide good contrast to background counts in the nodal basin; and it should have minimal pass-through effect to non-SLN nodes [7]. However, radiopharmaceuticals currently used for SLN detection in China, such as filtered ^99m^Tc-labeled sulfur colloid (SC), ^99m^Tc-labeled dextran (DX), or ^99m^Tc-human serum albumin (HSA), do not meet these criteria because of nonspecific uptake in non-SLNs, variable injection-site clearance, or both [8–10]. We propose that a rationally designed radiopharmaceutical will improve diagnostic performance when identifying SLNs.

The human B-lymphocyte-restricted differentiation antigen, Bp35 (CD20), is a cell surface non-glycosylated phosphoprotein that is overexpressed on malignant human B cells and normal B cells in the outer cortex of lymph nodes, particularly in the germinal centers [11]. Rituximab (Rituxan^®^, IDEC-C2B8) is a chimeric human–mouse monoclonal antibody (mAb) that is specific for CD20 and is used in the treatment of B cell non-Hodgkin lymphoma [12]. Thus, we hypothesized that ^99m^Tc-labeled rituximab (^99m^Tc-rituximab), once administrated into the interstitial space, would move by lymphatic flow, bind with the CD 20 molecules on B cells, and be retained in SLNs. This radiotracer, based on antigen–antibody-binding technology, should facilitate optimal sentinel lymphoscintigraphy.

In this study, we reported the synthesis and testing of ^99m^Tc-rituximab with the biochemical properties required for SLNs detection. The constant molecular weight and high antigen affinity of this compound produced a radiotracer with rapid injection-site clearance and low accumulation in distal lymph nodes. Finally, we assessed the value of lymphoscintigraphy and SLNB with^99m^Tc-rituximab for SLN detection in patients with breast cancer.

## RESULTS

### Preparation with ^99m^Tc-rituximab

The radiolabeling rate of ^99m^Tc-rituximab was determined to be 91.3% ± 1.1% (n = 5) at a specific activity of approximately 70 Mbq/μmol, using silica gel instant thin-layer chromatography (SG-ITLC). Purification of the ^99m^Tc-rituximab was performed by size-exclusion chromatography, and the final radiochemical purity of all ^99m^Tc-rituximab preparations was >99%. There was no appreciable loss in the radiochemical purity of ^99m^Tc-rituximab over 24 h when stored at room temperature, with the final levels of purity being 91% (unpurified) or 99% (purified). After incubating the ^99m^Tc-rituximab at 37°C with human serum for 24 h, the radiochemical purity decreased to approximately 78%.

### Immunoreactivity

**Figure 1:**
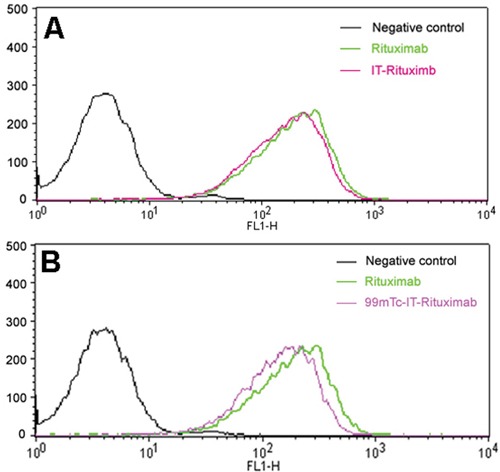
**A.** Cytofluorimetric comparison of unconjugated rituximab (green line) and IT-rituximab (pink line). **B.** Cytofluorimetric comparison of unconjugated rituximab (green line) and ^99m^Tc-IT-rituximab (purple line). The fluorescence intensities are presented as an overlay plot for identical antibody concentrations. The black line represents the results for a negative control, using human immunoglobulin.

The immunoreactivity of rituximab-based agents was analyzed by flow cytometric analysis (FCA), using human Burkitt's lymphoma Raji cells stably overexpressing CD20. Figure 1 illustrates the FCA results from Raji cells incubated with rituximab-based conjugates. Compared with the histogram of rituximab, histogram of 2-iminiothiolane-treated rituximab (IT-rituximab) showed no reduction of fluorescent intensity, suggesting an intact binding affinity. The binding capacity of ^99m^Tc-IT-rituximab was marginally impaired, as demonstrated by a shift of the histogram toward that of the Raji cells not incubated with the conjugate.

### SLN mapping and SLNB in mice

To test the suitability of the CD20 molecule as a target of the SLN agent, we subcutaneously injected ^99m^Tc-labeled rat-anti-mouse CD20 monoclonal antibody (^99m^Tc-anti-CD20 mAb) into the rear pads of bal b/c mice and compared it to nonspecific ^99m^Tc-labeled mouse immunoglobulin (Ig) G (^99m^Tc-IgG). The radiotracer was drained to popliteal SLNs and then to subiliac lymph nodes (secondary lymph node). The injecting activity percentages (%IAs) per lymph node sample at different time points after ^99m^Tc-anti-CD20 mAb or ^99m^Tc-IgG administration are compared in Table 1. The mean %IAs for SLNs with ^99m^Tc-anti-CD20 mAb were 2.13% ± 0.64%, 2.62% ± 1.25%, 2.26% ± 0.67%, 2.35% ± 0.69%, 1.62% ± 0.56%, 1.61% ± 0.81%, and 1.07% ± 0.07%, at 0.5 h, 1 h, 2 h, 4 h, 6 h, 8 h, and 24 h, respectively. The %IAs for each non-SLN decreased with time and was <0.5% at each time point. The blocking experiment, performed with a preinjection of excess anti-CD20 antibody at 4 h before study, showed a 50% reduction in SLN uptake. The ^99m^Tc-anti-CD20 mAb was excreted from the injection site gradually, and the percent of injected dose in the rear paws at 24 h was 12.62% ± 1.81%. The mean %IAs for the SLNs with ^99m^Tc-IgG were 2.21% ± 0.61%, 1.76% ± 0.06%, 1.87% ± 0.41%, 1.65% ± 0.17%, 1.23% ± 0.17%, 1.21% ± 0.11%, and 0.68% ± 0.12% at 0.5 h, 1 h, 2 h, 4 h, 6 h, 8 h, and 24 h, respectively. The percent of injected dose in the rear paw at 24 h was 7.46% ± 0.54%.

**Table 1:** Lymph node uptake and radioactivity of injection site at different times after intradermal injection of ^99m^Tc-labeled anti-CD20 antibody or ^99m^Tc-labeled mouse IgG into the left rear footpad of mice

Time	SLN (%IA)		NSLN (%IA)		IS (%IA)	
	Anti-CD20	IgG	Anti-CD20	IgG	Anti-CD20	IgG
30 min	2.13 ± 0.64	2.21 ± 0.61	0.46 ± 0.12	0.51 ± 0.08	66.73 ± 7.08	59.54 ± 8.62
1 h	2.62 ± 1.25	1.76 ± 0.06	0.35 ± 0.13	0.53 ± 0.03	49.18 ± 11.15	52.47 ± 9.82
2 h	2.26 ± 0.67	1.87 ± 0.41	0.33 ± 0.09	0.52 ± 0.12	39.12 ± 7.12	47.47 ± 2.20
4 h	2.35 ± 0.69	1.65 ± 0.17	0.37 ± 0.19	0.36 ± 0.15	29.04 ± 3.30	34.58 ± 0.71
4-h blocking	1.15 ± 0.25	1.53 ± 0.25	0.31 ± 0.04	0.30 ± 0.03	35.48 ± 1.99	33.99 ± 2.56
6 h	1.62 ± 0.56	1.23 ± 0.17	0.18 ± 0.01	0.26 ± 0.10	30.36 ± 1.44	28.90 ± 3.97
8 h	1.61 ± 0.81	1.21 ± 0.11	0.09 ± 0.05	0.26 ± 0.06	29.25 ± 6.93	12.83 ± 6.35
24 h	1.07 ± 0.07	0.68 ± 0.12	0.10 ± 0.04	0.18 ± 0.06	12.62 ± 1.81	7.46 ± 0.54

SLN, sentinel lymph node; NSLN, non-sentinel lymph node; %IA, percentage of injection activity, IS, injection site (3–5 mice/group); Anti-CD20, rat anti-mouse CD20 monoclonal antibody; IgG, mouse IgG.

**Figure 2:**
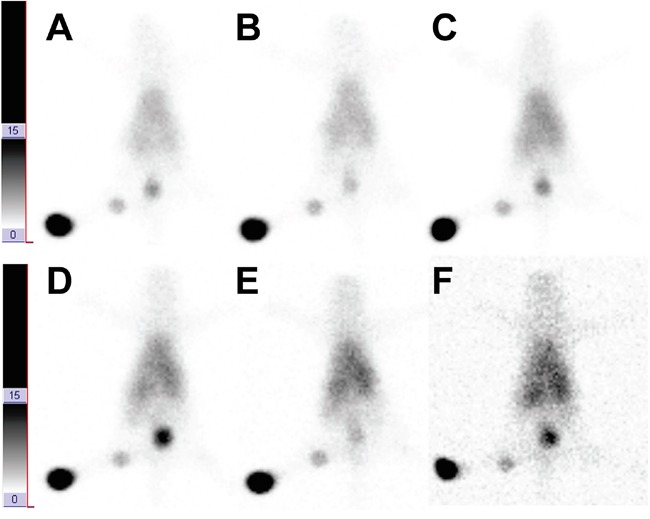
Dynamic sentinel lymphoscintigraphy at 30 min, 1 h, 2 h, 4 h, 8 h, and 16 h A–F after intradermal injection of ^99m^Tc-anti-CD20 antibody in the rear pad of bal b/c mice.

Figure 2, Supplementary Figure S1 and Figure S2 are representative of the imaging study. Thirty minutes after administration of ^99m^Tc-anti-CD20 mAb on the rear pad, the first-order popliteal lymph node was easily visualized. The radioactivity of SLNs was steady from 1–4 h postinjection and remained visible even after 24 h, and no second-order node or subiliac lymph node was detected. At 24 h after administration of the nonspecific ^99m^Tc-IgG, there was a faint impression of the node, presumably due to the flow of radioactivity through the lymph channel and passive accumulation within lymph fluid in the node; this process peaked at 1 h. A chain of lymph nodes was visualized 10 min after intradermally injecting ^99 m^Tc-SC and ^99m^Tc-DX on the rear pad (Supplementary Figure S1 and Figure S2).

### Biodistribution in mice

Biodistribution studies of ^99m^Tc-anti-CD20 mAb and^99m^Tc-rituximab in bal b/c mice were compared from 30 min to 24 h after the intradermal injection. Results were comparable for ^99m^Tc-anti-CD20 mAb and ^99 m^Tc-rituximab, as summarized in Table 2 and Table 3. The blood clearance was relatively slow because of the intradermal injection and large molecular weight (blood values were reduced by 60% from 30 min to 24 h). Radio conjugates were mainly excreted from the kidney and liver, and no accumulation was found in normal tissues.

**Table 2:** Biodistribution results of %IA/g of ^9m^Tc-labeled anti-CD20 antibody at 8h are same as those of ^99m^Tc-rituximab in bal/c mice (intradermal injection of the left rear footpad, %IA/g ± SD, n = 3-4)

Organ	30 min	1 h	2 h	4 h	4-h blocking	6 h	8 h	24 h
**Blood**	10.04 ± 2.11	11.20 ± 2.13	10.95 ± 3.23	9.41 ± 1.41	10.06 ± 0.60	8.69 ± 0.59	8.07 ± 0.05	4.08 ±0.15
**Heart**	1.98 ± 0.51	2.33 ± 0.06	2.65 ± 1.24	2.21 ± 0.49	2.09 ± 0.29	1.92 ± 0.09	2.38 ± 0.35	1.35 ± 0.09
**Liver**	3.55 ± 0.17	4.98 ± 2.17	4.61 ± 0.59	4.42 ± 1.06	5.45 ± 0.26	5.61 ± 1.70	5.95 ± 1.04	3.26 ± 0.21
**Spleen**	2.06 ± 0.35	2.49 ± 0.63	2.52 ± 0.54	2.44 ± 0.66	2.68 ± 0.29	2.64 ± 0.87	3.11 ± 0.14	2.06 ± 0.24
**Lung**	3.27 ± 0.29	3.49 ± 0.82	4.07 ± 1.44	3.07 ± 0.68	3.94 ± 0.77	3.40 ± 0.30	3.89 ± 1.19	1.97 ± 0.07
**Kidney**	8.07 ± 0.53	11.48 ± 1.09	11.34 ± 3.78	13.80 ± 1.29	13.94 ± 1.82	12.85 ± 2.92	13.49 ± 4.16	8.84 ± 0.20
**Stomach**	1.57± 0.54	1.92 ± 0.77	2.23 ± 0.75	3.00 ± 0.56	3.48 ± 0.90	2.55 ± 0.77	2.34 ± 0.41	1.07 ± 0.11
**Intestine**	0.88 ± 0.08	1.29 ± 0.77	1.70 ± 1.02	1.27 ± 0.26	1.71 ± 0.08	1.40 ± 0.11	1.56 ± 0.36	0.85 ± 0.16
**Muscle**	0.28 ± 0.10	0.50 ± 0.30	0.37 ± 0.13	0.27 ± 0.07	0.36 ± 0.08	0.30 ± 0.11	0.48 ± 0.09	0.35 ± 0.06
**Bone**	1.15 ± 0.73	0.57 ± 0.36	0.56 ± 0.16	0.93 ± 0.20	1.16 ± 0.28	1.53 ± 0.65	1.59 ± 0.31	1.23 ± 0.35

**Table 3:** Biodistribution results of ^99m^Tc-labeled rituximab in bal/c mice (intradermal injection of the left rear footpad, %IA/g ± SD, n = 3-4)

Organ	30 min	1 h	2 h	4 h	6 h	8 h	24 h
**Blood**	10.45 ± 1.06	11.19 ± 1.58	10.00 ± 0.37	7.71 ± 0.16	5.67 ± 026	4.88 ± 0.62	2.62 ± 0.65
**Heart**	1.50 ± 0.11	1.76 ± 0.64	2.25 ± 0.41	1.92 ± 0.17	1.32 ± 0.19	1.23 ± 0.19	0.78 ± 0.06
**Liver**	2.65 ± 0.17	3.03 ± 1.15	3.23 ± 0.13	2.58 ± 0.08	2.23 ± 0.29	2.31 ± 0.11	1.19 ± 0.27
**Spleen**	2.72 ± 0.26	3.23 ± 0.77	3.65 ± 0.29	2.77 ± 0.14	1.89 ± 0.49	2.24 ± 0.20	1.38 ± 0.23
**Lung**	2.84 ± 0.18	3.26 ± 0.71	3.40 ± 0.67	2.66 ± 0.26	2.49 ± 0.34	2.48 ± 0.26	1.30 ± 0.29
**Kidney**	5.35 ± 0.78	7.50 ± 1.11	11.44 ± 0.48	15.5 8± 0.79	15.30 ± 1.80	14.90 ± 2.05	9.73 ± 1.19
**Stomach**	0.79 ± 0.10	1.07 ± 0.46	1.05 ± 0.17	1.22 ± 0.28	1.57 ± 0.79	1.20 ± 0.14	0.59 ± 0.11
**Intestine**	0.64 ± 0.15	1.10 ± 0.59	1.46 ± 0.23	1.33 ± 0.12	1.17 ± 0.34	1.05 ± 0.12	0.65 ± 0.17
**Muscle**	0.24 ± 0.03	0.28 ± 0.04	0.33 ± 0.03	0.34 ± 0.12	0.31 ± 0.08	0.28 ± 0.02	0.24 ± 0.01
**Bone**	0.91 ± 0.18	0.87 ± 0.13	1.10 ± 0.27	1.11 ± 0.03	0.72 ± 0.04	0.80 ± 0.38	1.03 ± 0.94

### Dynamic SLN mapping in patients with breast cancer

**Figure 3:**
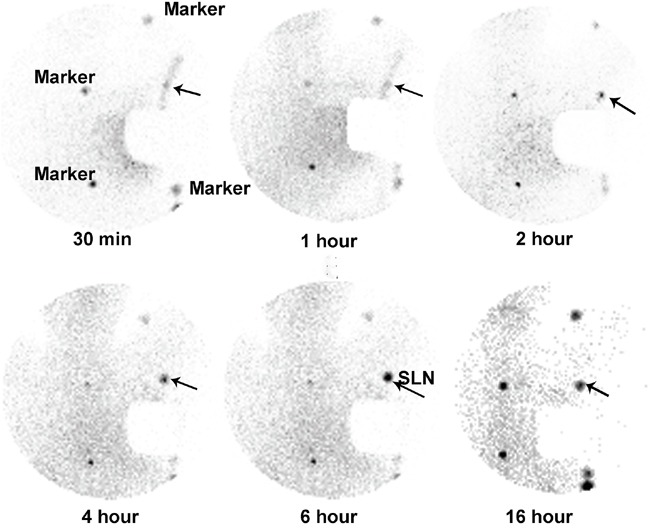
Dynamic sentinel lymphoscintigraphy at preselected time points after injecting ^99m^Tc-rituximab in a patient with a left breast cancer (Arrow: SLN).

The clinical characteristics of 12 patients who underwent dynamic scintigraphy are shown in Table 4. Tumors ≤ 2 cm were present in 10 patients, and tumors ≥ 2 cm but ≤5 cm were present in 2 patients; of these, 11 were invasive ductal carcinomas, and 1 was an invasive lobular carcinoma. Dynamic scintigraphy was obtained in the anterior view after the intradermal/intraparenchymal injection of ^99m^Tc-rituximab around the primary lesion at preselected times. The body contour was identified with the aid of a radioactive point source. Figure 3 shows an SLN visible in the left axilla of a patient with breast cancer. The radioactivity of the SLNs peaked at 6 h postinjection of ^99m^Tc-rituximab and could be visualized for 16 h; no non-SLN was detected over this time period. Among the 12 patients, 25 sentinel nodes were visualized after injecting the radiotracer, including 22 axillary nodes and 3 internal mammary nodes (2.1 sentinel nodes per patient, range 1–4). The number of nodes did not change between the 30 min and either the 16 h or 24 h scan. The pathways of the radiopharmaceutical and lymph fluid could be easily visualized in some of the images acquired at 30 min after injection. SLN metastasis to axillary nodes was also present in 1 of the 12 patients.

**Figure 4:**
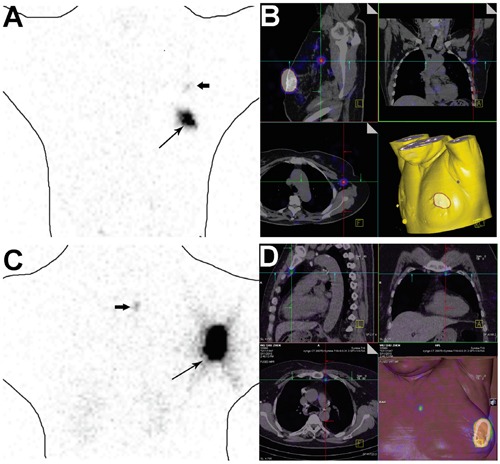
**A-B.** A 54-year old female with an infiltrating ductal carcinoma of the left breast. A sentinel lymph was localized in left axillary on sentinel planar lymphoscintigraphy (A) and SPECT/CT (B). **C-D.** A 37-year-old female with an infiltrating ductal carcinoma of the left breast. Sentinel planar lymphoscintigraphy (C) and SPECT/CT (D) identified an internal mammary node as the SLN.

**Table 4:** Clinical and histological features of 12 patients with breast cancer who underwent dynamic sentinel mapping

Patient	Age	Histology	TNM	Lesion		Mapping time (h)	SLN # (Mets)		ALND # (Mets)
				Location	Size (cm)		Axillary	IM	
1	51	IDC	T_1_ N_0_ M_0_	LUO	1.4 × 1.1	~16	3 (0)		28 (0)
2	36	ILC	T_1_ N_0_ M_0_	LUO	0.8 × 0.7	~18	1 (1)		12 (5)
3	58	IDC	T_1_ N_0_ M_0_	RUO	2.0 × 1.6	~17	1 (0)		16 (0)
4	57	IDC	T_1_ N_0_ M_0_	RU	1.3 × 1.2	~16	1 (0)		21 (0)
5	36	IDC	T_1_ N_0_ M_0_	RU	1.2 × 1.0	~16	2 (0)	1 (0)	25 (0)
6	39	IDC	T_1_ N_0_ M_0_	RU	1.1 × 0.8	~16	2 (0)		25 (0)
7	37	IDC	T_1_ N_0_ M_0_	RU	0.8 × 0.8	~16	2 (0)		22 (0)
8	51	IDC	T_2_ N_0_ M_0_	RUO	2.5 × 1.8	~16	2 (0)		17 (0)
9	39	IDC	T_1_ N_0_ M_0_	LUI	1.6 × 1.5	~16	2 (0)		25 (0)
10	52	IDC	T_1_ N_0_ M_0_	LL	1.6 × 1.5	~16	2 (0)	2 (0)	23 (0)
11	44	IDC	T_1_ N_0_ M_0_	LUO	1.4 × 1.3	~18	2 (0)		18 (0)
12	40	IDC	T_2_ N_0_ M_0_	LLI	2.8 × 2.5	~24	2 (1)		16 (0)

IDC, invasive ductal carcinomas; ILC, invasive lobular carcinoma; L, left; R, right; U, upper; L, low; O, outer; I, inner; IM, internal mammary; ALND, axillary lymph node dissection.

### SLN mapping and SLNB in patients with breast cancer

We enrolled 85 women with breast cancer for further investigation (Table 5). The average age was 47 years (range, 29–73 years), with 52.9% of patients (45/85) older than 50 years. Tumor size was ≤2 cm in 25 cases, ≥2 cm but ≤5 cm in 59 cases, and there was a ductal carcinoma *in situ* in 1 case. Of these, 75 had invasive ductal carcinomas, 4 had invasive lobular carcinomas, and 6 had other types of breast carcinoma.

**Table 5:** Clinical characteristics of 85 patients with breast cancer

Characteristics	Patients	Percentage
**Age (year)**		
≤50	40	47.1%
>50	45	52.9%
**Left or right side**		
Left	41	48.2%
Right	44	51.8%
**Primary tumor location**		
Upper inner quadrant	22	25.9%
Lower inner quadrant	6	7.1%
Upper outer quadrant	45	52.9%
Lower outer quadrant	12	14.1%
**Histopathological type**		
Invasive ductal carcinoma	75	88.2%
Invasive lobular carcinoma	4	4.7%
Other	6	7.1%
**T stage**		
Tis	1	1.2%
T1	25	29.4%
T2	59	69.4%

All 85 patients underwent planar lymphoscintigraphy, and nodes were identified in 82 cases. Single-photon emission computed tomography (SPECT)/computed tomography (CT) improved the anatomical identification of SLNs. Figure 4 shows that a marginal uptake was present in the axilla or internal mammary region, with SPECT/CT confirming SLN.

**Figure 5:**
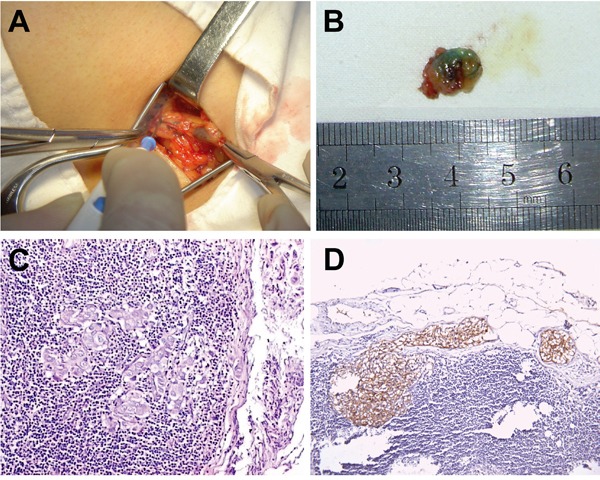
**A.**^99m^Tc-rituximab-guided SLNB, **B.** stained SLN, **C.** SLN with metastasis on hematoxylin and eosin staining, and **D.** SLN with micrometastasis on immunohistochemical staining with anti-CK19 monoclonal antibody.

The SLNs were identified in 96.5% (82/85) of patients when both blue dye and the intraoperative gamma probe were used. Stained and “hot” SLNs were excised by SLNB. Figure 5A and 5B showed the ^99m^Tc-rituximab-guided SLNB and the stained SLN. We identified 30 metastasis-positive SLNs (36.3%), including 24 positive cases identified on hematoxylin and eosin staining and 6 by immunohistochemistry (IHC) (Figure 5C and 5D). An SLN was the only metastatic lymph node in 18 of these 30 cases, and 1 case had a negative SLN. The sensitivity, specificity, and accuracy of SLNB were 96.8% (30/31), 100% (51/51), and 98.8% (81/82), while the false-negative rate, the negative predictive value, and the positive predictive value were 3.3% (1/31), 98.1% (51/52), and 100% (31/31), respectively.

## DISCUSSION

Our results demonstrate the favorable antigen-binding properties of a radiolabeled antibody-based lymphoscintigraphy agent. First, we demonstrated antigen specificity and affinity, as exhibited by the *in vitro* binding assay and the *in vivo* study. Then, the imaging and biodistribution data of mice were used to demonstrate the faster injection-site clearance and lower distal lymph node accumulation compared with nonspecific radiopharmaceuticals. Finally, clinical lymphoscintigraphy and biopsy data confirmed a high uptake of ^99m^Tc-rituximab in the SLNs of patients with breast cancer. These features are highly desirable in such an agent.

Imaging of SLNs has typically relied on two classes of agents: 1) radiolabeled particulates that bind to lymphoid tissue and 2) radiolabeled macromolecules that provide node images as they flow through the lymph node chain [13]. SLN imaging of breast cancer predominantly uses unspecific tracers ^99m^Tc-SC, ^99m^Tc-DX, and ^99m^Tc-HSA, and lymphoscintigraphy with these agents has important drawbacks [2, 14, 15]. The most frequently noted problem for ^99m^Tc-SC and ^99m^Tc-DX was that the timing of the radiotracer injection before surgery may significantly affect the number of nodes [16], and when multiple nodes are visualized, one cannot assume which is the sentinel node. Furthermore, the retention of radiocolloid in the injection site could obscure the SLNs, particularly those close to the injection site, because the large particles in the radiotracer may slow drainage or remain *in situ* [10]. For ^99m^Tc-HSA, it did not suffer from deficiencies described above; however, the accumulation in the SLNs gradually decreased with time, and sentinel lymphoscintigraphy and SLNB should be performed with 4 h, consistent with previous reports [17]. Thirdly, the particle sizes of nonspecific imaging agents are heterogeneous, and therefore, the number of particles administered varies and cannot be standardized for clinical practice. Lastly, ^99m^Tc-SC, ^99m^Tc-DX, and ^99m^Tc-HSA have not been approved by the CFDA, and current practice is based on institutional decisions.

A specific lymphoscintigraphy pharmaceutical, ^99m^Tc-tilmanocept, was approved by the U.S. FDA for SLN protocols for patients with breast cancer and melanoma in 2013 and for patients with head and neck squamous cell carcinoma in 2014 [18]. ^99m^Tc-tilmanocept is a receptor-based radiotracer that can specifically bind to mannose receptors (CD206), which are found in high concentrations on the surface of macrophages and dendritic cells [19]. This agent has demonstrated faster injection-site clearance than any nonspecific tracer, while retaining equivalent primary SLN uptake. Clinical data have showed an identification rate of 85%–97% and a sensitivity of 90%–100% when SLNB was followed by axillary lymph node dissection [20]. However, to date, ^99m^Tc-tilmanocept has not been approved by the CFDA, and no clinical trials have been published using this specific tracer in China.

Besides the need for ligand–receptor-specific binding, an antigen and antibody reaction also works with a lock-and-key mechanism. ^99m^Tc-rituximab is an antibody-based radiotracer that targets the CD20 antigen, overexpressing on the surfaces of B-lymphocytes in lymph nodes [21]. Theoretically, ^99m^Tc-rituximab may be injected into the interstice, from where it may drain to SLNs via the regional lymphatic system, and then specifically bind to CD20 in SLNs, where it is retained. To test this hypothesis, we established a mouse model and intradermally injected ^99m^Tc-labeled rat anti-mouse CD20 mAb into a rear pad. The ^99m^Tc-anti-CD20 mAb specifically bound to mouse CD20 molecules in the SLN, and the interactions between the ^99m^Tc-anti-CD20 antibody and CD20 enabled avid retention for up to 24 h, with a %IA of approximately 2.62% for SLNs, potentially limiting transmission to second-echelon nodes (to less than 0.5%). Because of the homogenous size (molecular weight, 150 kDa), ^99m^Tc-anti-CD20 mAb could exit the injection site gradually, with only 12.62% ± 1.81% of the radiotracer being retained *in situ*. These preclinical data showed the superior properties of ^99m^Tc-anti-CD20 mAb over unspecific tracers and proved that the CD20 molecule was the suitable target for the SLN imaging.

Because no consensus protocol exists for SLN assessment in breast cancer, we first performed the dynamic imaging in 12 patients to establish a standard protocol for good clinical practice. The dynamic imaging began as early as 5 min after administration of ^99m^Tc-rituximab and persisted for 16 to 24 h after the injection. Our data showed the SLNs of all 12 patients were visualized on the first imaging, and the accumulation of ^99m^Tc-rituximab in SLNs persisted for at least 16 h, with neither the number nor the location changing from the first to the last scan. Thus, the radiotracer injection before imaging and surgery could be performed within the wide range of time, and it is very convenient for clinician to choose time according to the practice.

The identification and false-negative rates are important safety parameters for SLNB. In 2005, guidelines from the American Society of Clinical Oncology stressed that a multidisciplinary team should aim to achieve a sentinel node identification rate of 85% with a false-negative rate of ≤5% before abandoning axillary dissection [22]. In our study, ^99m^Tc-rituximab-guided mapping and biopsy successfully identified at least one SLN in 82 of the 85 patients, giving an identification rate of 96.5% and a false-negative rate of 3.3%. These parameters were not only comparable to those of ^99m^Tc-tilmanocept (98.6% & 3.7%) [7, 23] but also superior to those of nonspecific agents [24–27]. No patient experienced adverse events that were considered as probably or definitely related to ^99m^Tc-rituximab by the investigators.

In this study, data demonstrated that SPECT/CT can be useful in several situations: 1) if SLN is not detected; 2) if extra-axillary lymph nodes are detected; 3) if an internal mammary lymph node is detected; 4) if a contamination is suspected; or 5) if a patient has a high body mass index, has undergone previous breast surgery, or has contralateral uptake [18, 28, 29].

In conclusion, ^99m^Tc-rituximab showed rapid injection-site clearance and promising localization ability for SLNs without non-SLN accumulation. Moreover, the bench data were successfully translated into clinical results for SLNB among patients with breast cancer. Efforts are now being made to conduct a trial in a larger cohort of patients with breast cancer and will be reported in our next study.

## MATERIALS AND METHODS

### Materials and cell line

Rituximab (Rituxan™) was purchased from Roche, Inc. (Shanghai, China). Rat anti-mouse CD20 monoclonal antibody (Anti-CD20 mAb, AISB12) was obtained from Abcam Co. (Cambridge, MA). Mouse IgG was purchased from Kangbao Biological product Co. Ltd (Shanxi, China). Triethanolamine, 2-iminiothiolane (2-IT), calcium glucoheptonate, and stannous chloride dehydrate were obtained from Sigma-Aldrich Co. (St. Louis, MO). TLC plates were obtained from EMD Chemicals (Gibbstown, NJ). ^99m^Tc-sodium pertechnetate, ^99m^Tc-labeled sodium colloid, and ^99m^Tc-labeled dextran from Beijing Atom High Tech Co. Ltd (Beijing, China). Human Burkitt's lymphoma cell Raji cells stably overexpressing CD 20 antigen were cultured as previously described [28].

### Preparation of the ^99m^Tc-labeled protein

Rituximab, Anti-CD20 mAb, and mouse IgG were purified from other excipients (Tween-80 and sodium citrate) by ultrafiltration. Then, 100 μg 2-IT was dissolved in a 1 mol/L triethanolamine buffer (solvent: 0.1 M, phosphate buffer; pH, 7.4) at a concentration of 1 mg/ml and incubated with 10 mg purified rituximab at 4°C for 45 min, with a chelator-to-antibody molar ratio of 20:1. The protein concentration was determined by ultraviolet absorbance at 280 nm. Next, 0.5 mg of IT-rituximab was mixed with 1 mg of calcium glucoheptonate and 10 μg of stannous chloride dehydrate (dissolved in 0.1 N HCl), followed by 370 MBq ^99m^Tc-sodium pertechnetate. The mixture was incubated at room temperature for 15 min and purified by size-exclusion chromatography with the saline. ^99m^Tc-labeled anti-CD20 mAb and ^99m^Tc-labeled mouse IgG were in the same way.

### Determination of radiochemical purity and serum stability

The radiolabeling efficiency of ^99m^Tc was assessed by silica gel instant thin-layer chromatography (SG-ITLC) through 2 developing systems (pyridine: acetone: distil water = 5:2:1; and acetone) using a Bioscan System 200 Imaging Scanner. In the first system, free pertechnetate and ^99m^Tc-glucoheptonate (trans-chelating agent) remained at the origin, while ^99m^Tc-labeled conjugates migrated to the front. In the second system, ^99m^Tc-labeled conjugate and ^99m^Tc-glucoheptonate remained at the origin, while free pertechnetate migrated to the front. The radiolabeling efficiency was calculated using the WinScan software (Bioscan, Washington, NW). The ^99m^Tc-rituximab in saline was stored at room temperature for 24 h, and a mixture of 3.7 MBq radiolabeled rituximab and 1 ml human serum (1:1) (Millipore) was incubated at 37°C for 24 h. Aliquots of ^99m^Tc-rituximab or the mixtures were sampled and subject to SG-ITLC at set times.

### FCA

Briefly, 0.3 ×10^6^ Raji cells were incubated in 100 μL of a culture medium containing 5 μg of rituximab conjugate. After 30 min on ice, the cells were washed once with 4 mL of media and then incubated on ice for an additional 30 min with fluorescein isothiocyanate-conjugated goat anti-human IgG Fc fragment antibody (Bethyl Inc., U.S.). After a final wash, cells were resuspended in ice-cold Hank's buffer. Mean fluorescence intensity was assessed with flow cytometry (FACS-Calibur, Becton Dickinson). As a positive control for specific binding, rituximab was tested at an equivalent molar concentration, and, as a negative control, cells were treated with human immunoglobulin.

### SLN mapping and biopsy in mice

All the animal studies complied with the Guidelines of the Health Science Center, Institutional Animal Care and Use Committee, Peking University. Groups of 3–5 female bal b/c mice (20 ± 0.5 g) were used for all experiments.

The bal b/c mice were injected subcutaneously in a rear pad with 1 μg of ^99m^Tc-labeled anti-CD20 (approximately 1.85 MBq) in 5 μL of saline solution. To determine the nonspecific uptake of SLN, a group of mice were preinjected with 50 μg of rat anti-mouse CD20 mAb in the rear pad 30 min before the experiment. The mice were then sacrificed at predetermined times. Before sacrifice, 30 μL of 0.25% Patent Blue (Sigma-Aldrich) in saline solution was administered to a footpad. Five minutes later, mice were anesthetized with 2.5% isoflurane and 0.4 L/min oxygen and then euthanized with carbon dioxide gas. Popliteal lymph nodes, subiliac lymph nodes, and the injection site (foot pad) were collected, and the radioactivity counts were determined with an auto-well gamma counter (LKB-Wallace Ltd., Finland). The %IAs of the SLNs, non-SLNs, and the injection site were calculated.

Comparison of SLN mapping was conducted between ^99m^Tc-anti-CD20 mAb, ^99m^Tc-labeled mouse IgG, ^99m^Tc-SC, and ^99m^Tc-DX. Radiotracer (1.85 MBq/5 μL) was subcutaneously injected into foot pads. Dynamic planar images of the mice were obtained while prone at different time points, using a SPECT equipped with a pinhole collimator.

### Biodistribution in mice

Bal b/c mice were subcutaneously injected in the rear pad with 1 μg, 5 μL, 0.15 MBq ^99m^Tc-labeled anti-CD20 or ^99m^Tc-labeled rituximab. The subcutaneous biodistributions in the rear pad were studied at 0.5, 1, 2, 4, 6, 8, and 24 h post injection. Organs of interest were collected, rinsed of excess blood, weighed, and counted by a γ-counter with the injection standards. The percentage of injected activity per gram of tissue (%IA/g) was calculated for each tissue.

### SLN mapping in patients with breast cancer

The SLN study in patients with breast cancer was approved by the Institutional Review Board of Peking University Cancer Hospital and performed in accordance with the ethical standards of the 1964 Declaration of Helsinki and its later amendments, or comparable ethical standards. We identified 85 patients with biopsy-proven clinical stage T_1-2_N_0_ breast cancer who were eligible for planar lymphoscintigraphy and SLNB; 2 patients were later recruited thereafter for imploring the advantage of a SPECT/CT scan in SLN mapping. In total, 85 patients underwent SLNB followed by axillary lymph node dissection.

Lymphoscintigraphy was performed after intradermal and peritumor administration of ^99m^Tc-rituximab (0.1–0.2 mg, 1–2 mCi/1 mL), using a SPECT or SPECT/CT scanner equipped with a pinhole or low-energy high-resolution parallel collimator (SIEMENS). Dynamic lymphoscintigraphy was done at 0.5 h, 1 h, 2 h, 4 h, 6 h, and 16 h postinjection in 12 patients (Table 1). In all other patients, anterior and supine lateral planar images were obtained between 3–18 h postinjection. A CT component was acquired in a non-contrast phase for anatomical localization (modulated 40 mAs, 120 kV, slice thickness 3 mm). A “hot spot” was considered to be a sentinel node and was marked on the skin.

### SLNB in patients with breast cancer

Shortly before surgery, 1.0 ml of Patent Blue (Sigma) was injected intradermally. The sentinel node was identified and harvested after careful detection of radioactivity with a gamma-ray detection probe (Neoprobe 1500 and 2000, Johnson & Johnson Medical, Hamburg, Germany). Histopathological assessment of the sentinel nodes included hematoxylin and eosin and IHC staining with anti-CK19 monoclonal antibody (Becton Dickinson, San Jose, Calif., USA).

### Statistical analyses

Data are expressed as the mean ± coefficient of variation. Results were statistically analyzed by one-way analysis of variance for in vivo studies, with the chi-square criterion for multiple comparisons. Differences were considered significant for a *P*-value less than 0.05.

## SUPPLEMENTARY FIGURES


