# Supplementary material for: Technetium-99m-labeled rituximab for use as a specific tracer of sentinel lymph node biopsy: a translational research study
Source: Oncotarget. 2016 May 26;7(25):38810–21. doi: 10.18632/oncotarget.9614 (PMC5122431; doi:10.18632/oncotarget.9614)
Supplement: Supplementary file 1 [file oncotarget-07-38810-s001.pdf]

Technetium-99m-labeled rituximab for use as a specific tracer of sentinel lymph node biopsy: a translational research study

SUPPLEMENTARY FIGURES

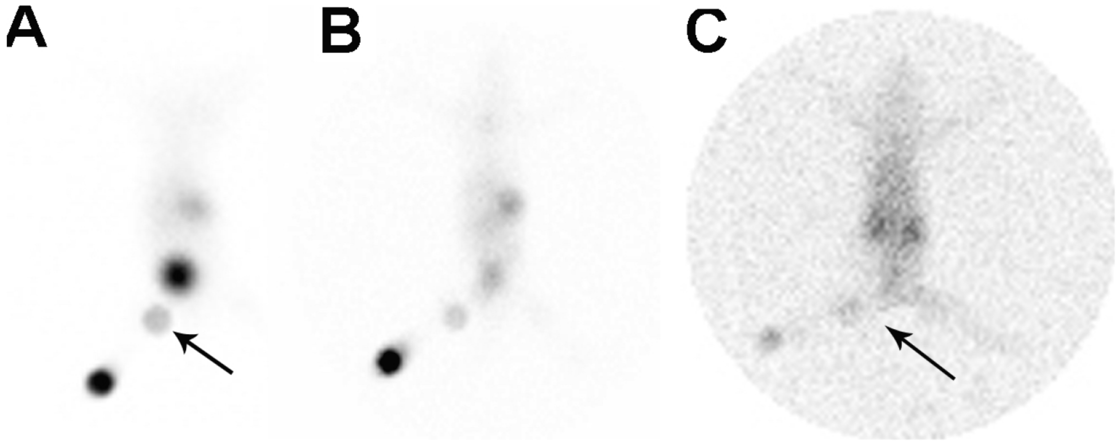

Supplementary Figure S1: A–C. Dynamic sentinel lymphoscintigraphy at 1, 4, and 24 h after intradermal injection of ^{99m}Tc-IgG antibody in the rear pad of bal/c mice (Arrow: sentinel lymph node).

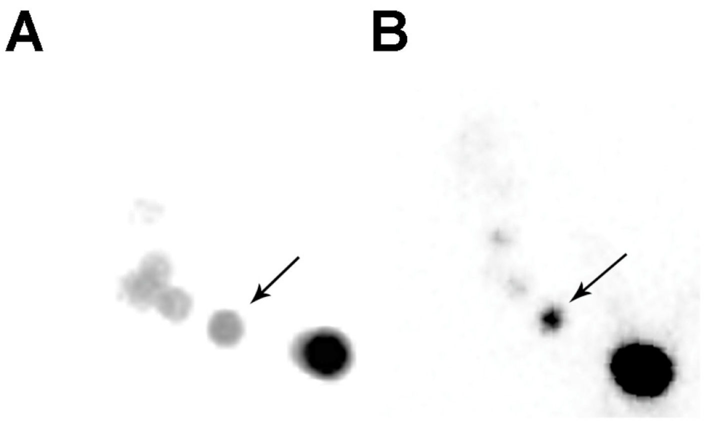

Supplementary Figure S2: Dynamic sentinel lymphoscintigraphy after intradermal injection of ^{99m}Tc-labeled dextran A. and ^{99m}Tc-labeled sulfur colloid **B.** in bal/c mice. A chain of lymph nodes were visualized 30 min after injecting the radiotracer in the rear pad of bal/c mice. (Arrow: sentinel lymph node).
